# Supplementary material for: Protective Mechanism of Polygonum perfoliatum L. Extract on Chronic Alcoholic Liver Injury Based on UHPLC-QExactive Plus Mass Spectrometry Lipidomics and MALDI-TOF/TOF Mass Spectrometry Imaging
Source: Foods. 2022 May 28;11(11):1583. doi: 10.3390/foods11111583 (PMC9179971; doi:10.3390/foods11111583)
Supplement: Supplementary file 1 [file foods-11-01583-s001.zip › foods-1718406-supplementary.pdf]

Supplementary information

Protective mechanism of *Polygonum perfoliatum* L. extract on chronic alcoholic liver injury based on UHPLC-QExactive Plus mass spectrometry lipidomics and MALDI-TOF/TOF mass spectrum imaging

Huaguo Chen [1, 2], Lei Peng [1, 2], Chao Zhao[1, 2], Zongwei Cai [3,*], Xin Zhou [1, 2,*]

1. Key Laboratory for Information System of Mountainous Areas and Protection of Ecological Environment, Guizhou Normal University, Guiyang 550001, China;

2. Guizhou Engineering Laboratory for Quality Control ＆ Evaluation Technology of Medicine, Guizhou Normal University, Guiyang 550001, China.

3. State Key Laboratory of Environmental and Biological Analysis, Department of Chemistry, Hong Kong Baptist University, Hong Kong SAR, China

Corresponding author

Xin Zhou: Guizhou Normal University, 116 Baoshan North Rd., Guiyang, 550001, China, *Tel.: +86-851-8669-0018; Fax: +86-851-8669-0018

E-mail address: alice9800@sina.com

Zongwei Cai: Hong Kong Baptist University, Hong Kong SAR, China

E-mail address: zwcai@hkbu.edu.hk

Contents

Figure S1 Total ion chromatograms (TIC) of compounds detection in *P. perfoliatum* extract 3

Table S1 Compounds identified by UHPLC-MS in negative ion mode 4

Table S2 MS/MS fragmentation of each identified compound 5

Figure S1 Total ion chromatograms (TIC) of compounds detection in *P. perfoliatum* extract

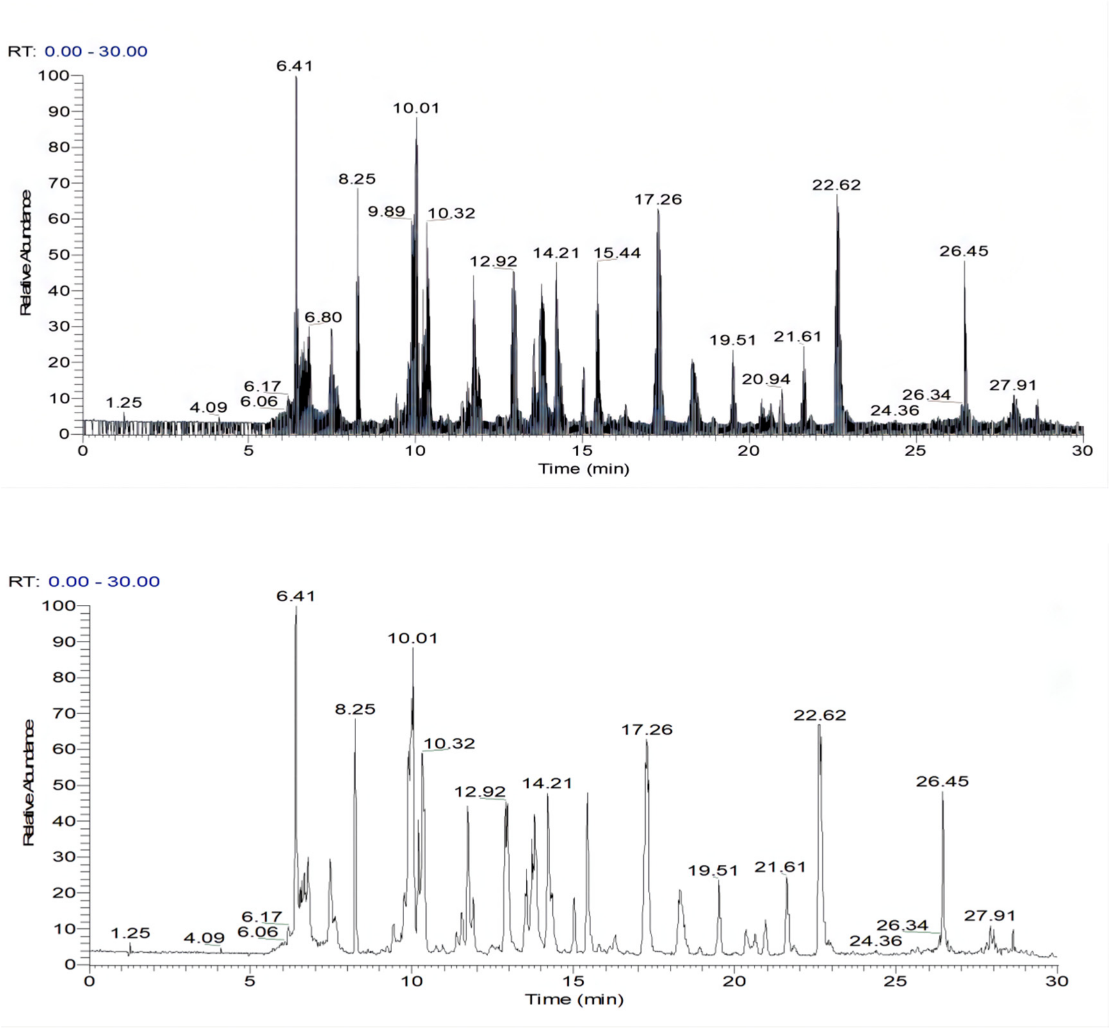

Table S1 Compounds identified by UHPLC-MS in negative ion mode

No.	t/min	Compound	Molecular formula	Estimated value (m/z)	Error	Secondary mass spectrometry (m/z)
1	6.17	Gallocatechin	$C_{15}H_{14}O_7$	305.7069	1.98	287.1098, 261.0209, 219.3217, 179.0732, 167.1120
2	6.41	Catechinic acid	$C_{15}H_{14}O_6$	289.5927	4.32	245.4611, 203.1266, 179.3412, 151.9015
3	8.25	Chlorogenic Acid	$C_{16}H_{18}O_9$	351.1774	2.19	191.1088
4	10.01	Epicatechin	$C_{15}H_{14}O_6$	288.7415	1.02	245.6725, 203.0728, 179.1927, 151.0307
5	10.32	Sanitol-*7-O*-rhamnose-*3-O*-glucoside	$C_{27}H_{30}O_{15}$	596.4124	-0.39	284.1092, 285.0802
6	12.92	Rutin	$C_{27}H_{30}O_{16}$	607.1754	1.64	300.1011, 301.2016
7	14.21	Kaempferol-*3-O*-neocyanin	$C_{27}H_{30}O_{15}$	592.122	3.22	284.2922, 285.1650
8	17.26	Isoquercitrin	$C_{21}H_{20}O_{12}$	462.9188	2.55	300.1213, 301.2090
9	19.51	Kaempferol rutin	$C_{27}H_{30}O_{15}$	591.5722	4.11	284.3731, 285.4232
10	21.61	Quercitrin	$C_{21}H_{20}O_{11}$	448.2144	2.68	300.2901, 301.2884
11	22.62	Quercetin	$C_{15}H_{10}O_7$	301.9162	3.77	273.1736, 229.8021, 179.0902, 151.1877
12	26.46	Kaempferol	$C_{15}H_{10}O_6$	284.2505	2.89	255.2334, 227.0519, 181.2976

Table S2 MS/MS fragmentation of each identified compound

NO.	Metabolite	Formula	Measured m/z	MASS accuracy (ppm)	Secondary mass spectrometry (m/z)
1	Stearoyl-L-Carnitine	$C_{25}H_{49}NO_4$	426.3421	-2.03	85.0100, 60.0182, 84.0147, 86.0118
2	6-Aminohexanoate	$C_6H_{12}NO_2$	131.3162	-0.79	115.0772, 114.0931, 97.0673, 69.0712
3	Linoleic acid	$C_{18}H_{32}O_2$	280.3325	-0.91	264.2402, 245.2264, 97.1009
4	PC(16:0/18:1)	$C_{42}H_{82}NO_8P$	703.4683	1.49	506.4024, 406.1626, 281.1215, 224.0020
5	LPE(20:3)	$C_{25}H_{46}NO_7P$	503.3612	-0.95	440.2333, 214.0486, 152.9958, 78.9591
6	Bexarotene	$C_{24}H_{28}O_2$	348.5070	-0.71	279.1367, 251.1056, 227.1778
7	Ginkgolide B	$C_{20}H_{24}O_{10}$	424.4803	1.35	368.1410. 367.1388, 125.0237, 113.0234
8	Abietic acid	$C_{20}H_{30}O_2$	302.2624	-0.97	257.2265, 149.1325, 123.1169, 121.1012
9	SM(d18:1/18:0)	$C_{41}H_{83}N_2O_6P$	730.5561	-1.06	184.0749, 104.1077, 86.0971
10	SM(d18:0/16:0)	$C_{39}H_{81}N_2O_6P$	704.4763	-0.11	619.4890, 464.3328, 239.2369, 74.0964
